# Diagnostic Accuracy of AI-Assisted Focused Cardiac Ultrasound (FOCUS) in Primary Care

**DOI:** 10.3390/healthcare13212726

**Published:** 2025-10-29

**Authors:** Mihai-Sorin Iacob, Nilima Rajpal Kundnani, Abhinav Sharma, Andrei Iacob, Anca-Raluca Dinu, Simona Ruxanda Dragan

**Affiliations:** 1 Doctoral School, “Victor Babes” University of Medicine and Pharmacy, 3000041 Timisoara, Romania; 2 University Clinic of Internal Medicine and Ambulatory Care, Prevention and Cardiovascular Recovery, Department VI—Cardiology, “Victor Babes” University of Medicine and Pharmacy, 3000041 Timisoara, Romania; 3 Research Centre of Timisoara Institute of Cardiovascular Diseases, “Victor Babes” University of Medicine and Pharmacy, 3000041 Timisoara, Romania; 4 Faculty of Medicine, “Vasile Goldis” University of Vest, 310025 Arad, Romania; 5 Department XVI, Medical Recovery, “Victor Babes” University of Medicine and Pharmacy, 300041 Timisoara, Romania; 6 Research Center for Assessment of Human Motion and Functionality and Disability, “Victor Babes” University of Medicine and Pharmacy, Eftimie Murgu Square, No. 2, 300041 Timisoara, Romania; 7 ”Pius Brinzeu” Emergency Clinical County Hospital, Bld Liviu Rebreanu, No. 156, 300723 Timisoara, Romania

**Keywords:** point-of-care ultrasound (POCUS), cardiovascular diseases, strategies, artificial intelligence, left ventricular ejection fraction (LVEF)

## Abstract

**Background**: Focused cardiac ultrasound (FOCUS) can augment the cardiac exam in primary care but is operator-dependent. We evaluated the diagnostic performance of artificial intelligence-assisted FOCUS (AI-FOCUS) performed by family physicians against cardiologist-performed echocardiography. **Methods**: This research is a prospective cross-sectional study in primary care; family physicians performed conventional FOCUS and AI-FOCUS, with cardiologist-performed echocardiography within 24 h as the reference standard. The primary outcomes were accuracy, sensitivity/specificity, and agreement (κ). **Results**: AI-FOCUS achieved 94.33% accuracy (95% CI 93.15–95.35), 89.91% sensitivity, and 96.49% specificity, with excellent agreement compared to cardiologists (κ = 0.88). Among the confirmed abnormalities (32.9% of participants), valvular disease was most frequent (42%), followed by reduced LVEF < 50% (28%) and pericardial effusion (12%). In multivariable analysis, AI-assisted LVEF < 50% (OR = 6.05, *p* < 0.0001) and valvular abnormalities (OR = 4.05, *p* < 0.0001) were strong predictors of cardiac pathology. **Conclusions**: AI-FOCUS performed by trained family physicians showed high diagnostic accuracy and excellent agreement with blinded cardiologist-performed echocardiography for detecting LVEF < 50%, screening-level valvular abnormalities, and pericardial effusion, supporting its use for early detection and triage in primary care. Its ease of use and reproducibility suggest value in settings with limited access to cardiology.

## 1. Introduction

Cardiovascular diseases (CVDs) remain the leading cause of global mortality, accounting for ~32% of all deaths, according to the World Health Organization (WHO) [1]. In primary care, delayed or missed diagnoses of cardiac conditions contribute to up to 30% of preventable cardiac hospitalizations [2,3]. These conditions often progress insidiously, with subtle clinical signs that may be overlooked during routine consultations. As a result, patients frequently present only after decompensation or irreversible structural damage has occurred [4].

Primary care is crucial for reducing cardiovascular burden and disease worldwide, as it represents the front line of medicine, even in areas with relatively lower incomes and diagnostic means. Within PHC, population-level risk assessment, hypertension and diabetes management, and nurse- or pharmacist-led adherence programs reduce incident CVD and hospitalizations, particularly when combined with guideline-based prevention pathways and community screening. Examples include systematic BP and diabetes screening during routine visits and opportunistic atrial fibrillation checks, which improve detection and timely referral from PHC. Embedding simple imaging tools within PHC workflows can further accelerate triage and reduce missed pathology. WHO and ESC have declared that, in certain populations, primary care-based interventions have shown to reduce CVD mortality by 20% [1,5]. Therefore, it is paramount that we invest as much as possible in cheap, easy-to-use and practical means of diagnosis and triage in primary care.

Traditional physical examinations lack the sensitivity to reliably detect many structural cardiac abnormalities, especially in early or subclinical stages [6]. While the clinical exam remains a cornerstone of cardiac assessment, its diagnostic accuracy is highly variable and dependent on clinician experience [7]. Moreover, key signs may be absent or misleading in early disease, leading to under-recognition of important cardiac conditions [8].

Focused Cardiac Ultrasound (FOCUS) has emerged as a valuable bedside tool to enhance diagnostic capabilities. As a limited but goal-directed ultrasound exam, FOCUS allows clinicians to rapidly assess cardiac structures and function with immediate clinical relevance [9]. Its utility in triage, heart failure management, and the assessment of volume status is well documented [10]. However, its adoption in primary care remains limited, largely due to operator dependency and variability in image interpretation [11,12]. For example, East et al. reported a sensitivity of only 78% for non-specialist-performed FOCUS without AI support [13]. These barriers are particularly pronounced among non-specialist users, such as family physicians, who may lack formal echocardiographic training and experience. This has led to concerns regarding the reproducibility and diagnostic accuracy of FOCUS when performed outside cardiology or emergency settings [14].

The AI platform uses automated image segmentation and speckle-tracking algorithms to quantify LVEF and detect wall motion abnormalities. Recent advancements in artificial intelligence (AI), particularly in automated left ventricular ejection fraction (LVEF) quantification and speckle-tracking strain analysis, offer promising solutions to these challenges [15,16]. AI-driven tools have demonstrated the potential to reduce inter-observer variability and enhance the precision of measurements. By automating image analyses, AI can help bridge the skill gap in primary care and allow larger access to high-quality cardiac imaging. This has shown to be useful even in areas where cardiac expertise is required but not always available, such as non-expert oncologists requiring cardiac clearance before starting chemotherapy, in which case AI-assisted Point-of-Care Ultrasound (POCUS) showed excellent correlation with the one performed by a cardiologist [17].

This study aimed to evaluate the diagnostic accuracy of AI-assisted FOCUS performed by family physicians in a real-world primary care population. Our hypothesis was that AI integration could standardize assessments and improve early detection of cardiac pathology. By examining the performance of AI-augmented FOCUS in a realistic clinical environment, our research seeks to assess the possibility of the implementation of point-of-care cardiac imaging in general practice.

This study provides the first large-scale, prospective primary-care validation of AI-assisted FOCUS across multiple cardiac pathologies, within a structured training framework for family physicians and with blinded, cardiologist-performed echocardiography as reference, including an exploratory analysis of downstream resource use.

## 2. Methods

### 2.1. Study Design and Population

We conducted a prospective, observational cross-sectional study at the Timis County Center in Romania, a country with one of the highest overall cardiovascular mortalities in Europe [18]. The unit of analysis was the patient-level binary classification ‘any target cardiac pathology present vs. absent’ by AI-FOCUS and by the cardiologist reference. By focusing on this dual inclusion criterion, of quantified risk scores and clinical suspicion, we aimed to simulate the real-world diagnostic dilemmas often encountered in primary care. On the other hand, patients with known prior cardiovascular disease, morbid obesity (BMI ≥ 40 kg/m^2^), severe pulmonary disease or other causes of potential inadequate acoustic windows were excluded, in order to avoid confounding from pre-established diagnoses and to ensure technical feasibility of image acquisition. For internal stability assessment of diagnostic estimates (no model fitting), we performed a random 70/30 split-sample analysis (n = 1246/n = 534) and five-fold stratified cross-validation at the patient level. These procedures assessed reproducibility of performance metrics and did not involve training, tuning, or modifying the proprietary AI model. 

### 2.2. FOCUS Protocol

All FOCUS examinations were performed by family physicians trained through a standardized 6-month curriculum. This training was designed to establish competency in cardiac image acquisition and interpretation, therefore emphasizing reproducibility. Training included a 6-month curriculum with supervised hands-on scanning and formal competency assessments. Training comprised didactic seminars, supervised scanning with >50 documented patient examinations, and a competency assessment covering image acquisition (standard views) and interpretation (LVEF, basic valvular assessment, pericardial effusion). Trainees maintained a logbook and were signed off by echocardiography-credentialed faculty. Each patient underwent both conventional FOCUS and AI-assisted FOCUS using a Sonoscape-P60 ultrasound system equipped with the Wis+ AI platform [19]. Performing both modalities allowed for a direct comparison of diagnostic performance, isolating the added value of AI without altering human medical experience. This curriculum aimed to ensure safe, standardized acquisition and screening-level interpretation—not specialist-level competency; the reference standard remained blinded cardiologist echocardiography.

Target conditions and standardized measurements. All enrolled patients underwent the same five-view FOCUS protocol and uniform AI processing. The prespecified target conditions were as follows:

(1) Left-ventricular systolic dysfunction, defined as an LVEF < 50% (AI-computed Simpson’s biplane; binary abnormal/normal);
(2) Valvular abnormalities (screening-level identification of ≥mild regurgitation or suspected stenosis on 2D/Doppler, recorded as present/absent and valve involved);
(3) Pericardial effusion (any echo-free space recorded as present/absent).

For every participant, we recorded image acquisition adequacy (pass/fail) and AI outputs. Cardiologist reference echocardiography applied ASE/EACVI criteria to the same endpoints and involved blinding to the FOCUS/AI results.

All three endpoints were assessed systematically in every patient.

The FOCUS protocol included five standardized acoustic views:

- Subxiphoid: assessment for pericardial effusion and inferior vena cava (IVC) collapsibility to evaluate pericardial and volume status [20]
- Parasternal long and short axis: evaluation of left and right ventricular dimensions, global and regional wall motion, and gross valvular morphology
- Apical four-chamber: AI-assisted LVEF quantification using Simpson’s biplane method, a guideline-recommended approach for volumetric assessment [21]
- Subcostal (IVC assessment): estimation of intravascular volume status via respiratory variability [22]

Thus, three assessments were obtained per patient (conventional FOCUS; AI-FOCUS; reference cardiologist echocardiography) using the same device; the cardiologist was blinded to FOCUS/AI results.

AI integration and outputs: Cine loops from standard views were automatically forwarded to the Wis+ module, which applies a convolutional neural network for endocardial border segmentation and computes LVEF (Simpson’s biplane). When LVEF was <50%, the system flagged an abnormal result. The module also provided speckle-tracking-based wall-motion support and Doppler-aided valvular screening. The family physician reviewed and recorded the AI output but did not alter it. The authors did not modify or retrain the proprietary model. See Supplementary Table S2 for model-level details. The Wis+ AI platform provided automated LVEF measurements and speckle-tracking [22] echocardiography, which tracks myocardial motion across the cardiac cycle to enhance the precision of assessment of kinetic abnormalities. Doppler flow patterns supported detection of valvular pathologies [23], adding hemodynamic context to morphological findings.

The study used a Sonoscape-P60 system with the Wis+ AI module in a European setting. We evaluated clinical performance only and made no claims regarding regulatory clearance in jurisdictions outside our study site. The cardiologist reference exam did not rely on AI outputs and was performed per ASE/EACVI standards. Implementation in other regions (e.g., the U.S.) should follow local regulations/FDA requirements; our study did not seek or establish device certification.

### 2.3. Reference Echocardiography

To establish diagnostic validity, a blinded cardiologist, unaware of the FOCUS results and patient clinical history, performed comprehensive reference echocardiography on the same ultrasound system (Sonoscape-P60: Sonoscape Medical Corp., Shenzhen, China) within 24 h of each FOCUS exam, but without using the AI output for interpretation. This approach minimized inter-device variability and potential temporal changes in cardiac status. All measurements adhered strictly to American Society of Echocardiography (ASE) and European Association of Cardiovascular Imaging (EACVI) guidelines, ensuring standardization and clinical relevance [24,25].

**Figure 1:**
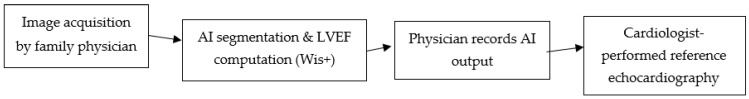
Workflow of AI-assisted FOCUS in primary care. Cine loops acquired by the family physician are processed by the Wis+ AI module for automated LV border detection and LVEF computation; physicians record AI outputs before blinded cardiologist-performed echocardiography within 24 h. Arrows are equal length and slope for visual clarity only; they do not encode timing or magnitude.

Figure 1 shows a schematic workflow of AI-assisted FOCUS:

### 2.4. Statistical Analysis

Diagnostic performance was assessed through sensitivity, specificity, positive predictive value (PPV), and negative predictive value (NPV), calculated using MedCalc v23.1. Agreement between FPs and cardiologists was measured with Cohen’s kappa (κ) and 95% confidence intervals (CI), reflecting inter-rater reliability across categorical findings. Logistic regression models, performed in SPSS v28, were used to identify independent predictors of cardiac pathology, incorporating demographics, cardiovascular risk factors, and key ultrasound parameters [26]. In addition to sensitivity, specificity, and κ statistics, ROC curves were generated for LVEF < 50% and valvular abnormalities. Five-fold cross-validation was applied to assess the robustness of diagnostic performance, with results remaining consistent across folds. Logistic regression models also adjusted for imbalance by including covariates such as age, sex, and risk factors. Five-fold stratified cross-validation was conducted at the patient level (stratified by the reference standard prevalence). For each fold, we computed sensitivity, specificity, PPV/NPV, accuracy, κ, and ROC metrics on the held-out fold while no model training or threshold tuning was performed. We report the overall estimates on the full dataset and verified that fold-wise results were consistent.

Metric definitions (patient-level, any-pathology endpoint). With the cardiologist exam as reference: TP = AI positive and reference positive; FP = AI positive and reference negative; TN = AI negative and reference negative; FN = AI negative and reference positive.

Sensitivity = TP/(TP + FN); Specificity = TN/(TN + FP); PPV = TP/(TP + FP); NPV = TN/(TN + FN); Accuracy = (TP + TN)/N; κ computed for binary agreement with 95% CIs.

For this study: TP = 526, FP = 42, TN = 1153, FN = 59 (N = 1780), which yield Accuracy 94.33%, Sensitivity 89.91%, Specificity 96.49%, PPV 92.61%, NPV 95.13%. 

## 3. Results

### 3.1. Participant Characteristics

The study enrolled a total of 1780 patients aged 40–75 years, all presenting either high cardiovascular risk (SCORE2-OP ≥ 10%) or symptoms suggestive of cardiac pathology (e.g., dyspnea, chest pain) [27], from community-based primary care practices across Timis County. The cohort had a mean age of 62 ± 9 years and a slight male predominance (54%). All participants met the inclusion criteria of either high estimated cardiovascular risk (SCORE2-OP ≥ 10%) or clinical symptoms suggestive of cardiac pathology (most commonly dyspnea or chest discomfort). Cardiac abnormalities, as confirmed by reference echocardiography, were detected in 585 individuals, representing a prevalence of 32.9% (95% CI: 30.7–35.1%).

### 3.2. Diagnostic Performance of AI-FOCUS

As detailed below in Table 1 and illustrated in Figure 1, the AI-enhanced modality achieved an overall accuracy of 94.33% (95% CI: 93.15–95.35), with a sensitivity of 89.91% (95% CI: 87.18–92.23) and specificity of 96.49% (95% CI: 95.28–97.46). Positive predictive value (PPV) and negative predictive value (NPV) were similarly robust at 92.61% (95% CI: 90.29–94.41) and 95.13% (95% CI: 93.88–96.14), respectively.

Agreement between AI-FOCUS and cardiologist assessments was excellent, with Cohen’s kappa = 0.88 (95% CI: 0.81–0.95), indicating near-perfect inter-rater reliability.

### 3.3. Distribution of Detected Cardiac Pathologies and Comorbidities

Among the 585 individuals with confirmed cardiac abnormalities, valvular disease was the most frequently observed pathology, affecting 42% of cases (n = 246). Mitral regurgitation was the predominant pathology within this category. AI algorithms automatically quantified reduced left ventricular ejection fraction (LVEF < 50%) in 28% of cases (n = 164), while pericardial effusion, often subtle and easily missed, was identified in 12% (n = 70), primarily through the subxiphoid acoustic window. The values are shown below in Table 2.

Misclassifications were most common in borderline cases, such as mild valvular regurgitations (particularly trivial–mild mitral regurgitation), borderline reductions in LVEF (45–50%), and small pericardial effusions (<5 mm). These cases were frequently underestimated or missed by AI-FOCUS, whereas cardiologist echocardiography provided more precise differentiation.

**Figure 2:**
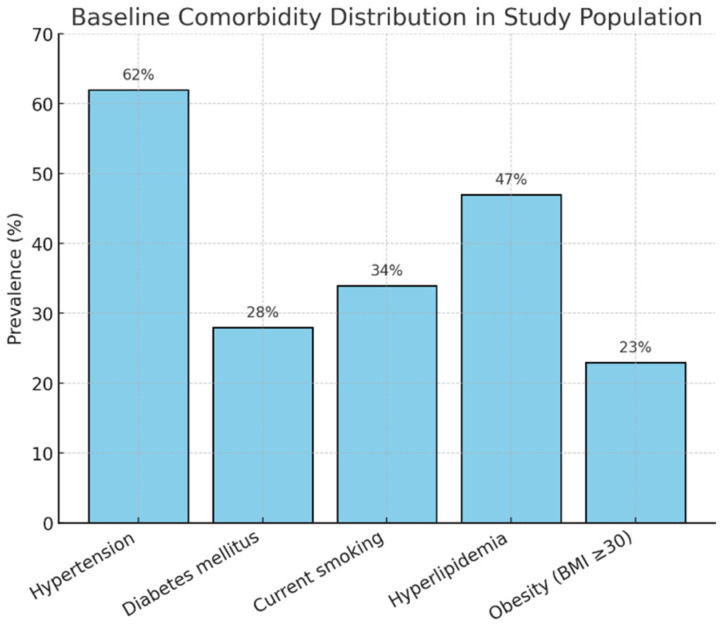
Comorbidity distribution.

Comorbidities were distributed as seen in Figure 2.

### 3.4. Predictors of Cardiac Pathology

Multivariate logistic regression identified four significant predictors for the presence of cardiac pathology. The four most significant independent variables are described below in Table 3.

### 3.5. Subgroup Analyses

Subgroup analyses were performed to assess how cardiac pathology prevalence varied by age, sex, and cardiovascular risk strata.

#### 3.5.1. Cardiac Pathology by Age Group

Cardiac pathology prevalence increased progressively with age. The highest prevalence was observed in patients aged 60–69 years (40.6%). Table 4 shows the prevalence of cardiac pathology in each studied age group.

#### 3.5.2. Cardiac Pathology by Gender

A modest sex-based difference was noted, with cardiac pathology more prevalent in male patients (35.4%) compared to female patients (29.9%). Table 5 shows the distribution and comparison between genders.

#### 3.5.3. Cardiac Pathology by SCORE2-OP Risk Strata

The SCORE2-OP (Older Persons) is a European Society of Cardiology (ESC) risk assessment model that estimates the 10-year risk of fatal and non-fatal cardiovascular disease (CVD) in adults over the age of 70 without a history of prior CVD or diabetes. It uses factors such as sex, age, smoking status, blood pressure, and cholesterol levels to determine an individual’s risk within specific geographical regions. Higher cardiovascular risk, as reflected by SCORE2-OP strata, correlated with higher prevalence of cardiac pathology. Table 6 shows the incremental increase in the prevalence of pathology correlating with a higher risk strata.

**Figure 3:**
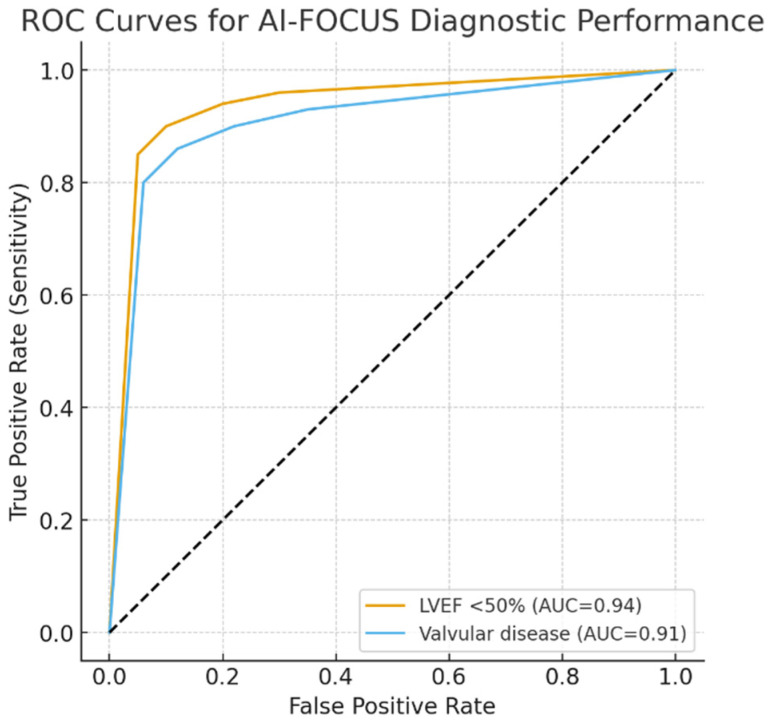
ROC curves for AI-FOCUS diagnostic performance. ROC, receiver operating characteristic; AUC, area under the curve. Dotted line: mid-line of chart.

We performed ROC curves for AI-FOCUS in detecting LVEF < 50% (AUC 0.94, 95% CI 0.92–0.96) and valvular abnormalities (AUC 0.91, 95% CI 0.88–0.93). Five-fold cross-validation demonstrated consistent AUC values across subsets, supporting robustness and generalizability (Figure 3).

**Figure 4:**
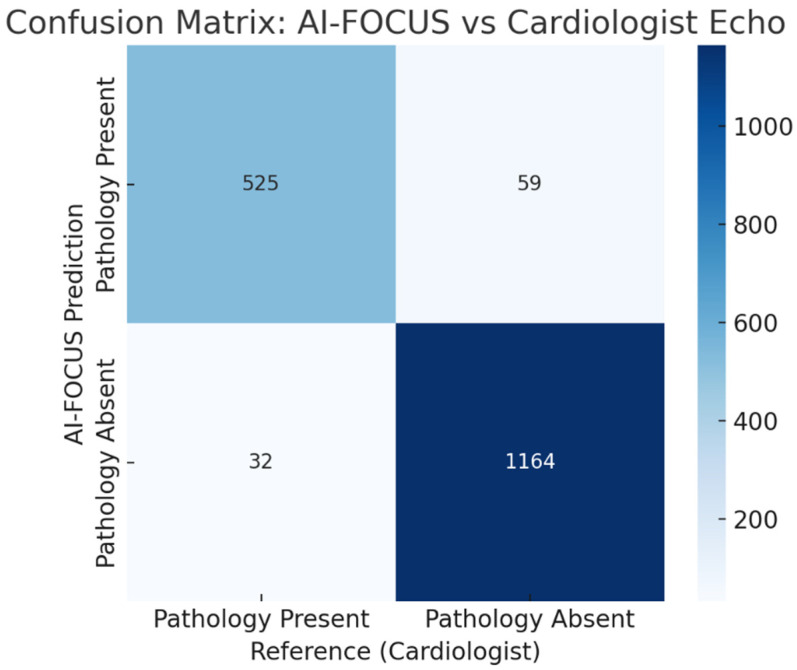
Confusion matrix comparing AI-FOCUS vs. blinded cardiologist echocardiography for the any-pathology endpoint at the patient level (N = 1780; TP = 526, FP = 42, TN = 1153, FN = 59). Misclassifications concentrated in borderline LVEF (45–50%) and mild valvular regurgitation.

Confusion matrix comparing AI-FOCUS vs. cardiologist echocardiography. Misclassifications concentrated in borderline LVEF and mild valvular regurgitation. (Figure 4).

## 4. Discussion

(Note: Supplementary Table S1 summarizes model features (population, training, AI platform), performance results, misclassification conditions, and limitations compared to prior studies.)

### 4.1. Clinical Implications

The clinical utility of Focused Cardiac Ultrasound (FOCUS) is gaining traction in primary care as a valuable tool for physical examination. Our findings suggest that the integration of AI into FOCUS technology represents a significant advancement in this evolving field. Specifically, AI-assisted FOCUS demonstrated a high level of diagnostic accuracy and very good inter-rater reliability when compared to protocols carried out by trained, human professionals. Moreover, this performance substantially exceeds that reported in earlier studies using non-AI FOCUS in non-specialist hands (78% sensitivity reported by Labovitz et al. [28]. Unlike prior smaller studies focused on single outcomes or specialist settings, our results derive from a large primary-care cohort with multiple outcomes and blinded validation.

The integration of AI into FOCUS protocols is of paramount importance because it allows general practitioners (GPs) to standardize echocardiographic cardiac measurements, especially left ventricular ejection fraction (LVEF) and valvular assessments, therefore ruling out operator dependency. Our statistics have shown that an AI-assisted LVEF < 50% emerged as the strongest independent predictor of pathology (OR = 6.05), findings which align with emerging evidence from other clinical environments. A multicenter prospective study by LoPresti et al. [29] reported the similarly high reproducibility of AI-assisted LVEF estimation when used by both beginner and experienced operators, with an intraclass correlation coefficient exceeding 0.90 and a near-perfect agreement on identifying abnormal LVEF (<50%) (AUC 0.98, sensitivity 92.8%, specificity 92.3%).

Looking at specific patient groups helped us better understand where AI-assisted FOCUS can be most useful. As expected, the rate of cardiac problems went up with age and higher cardiovascular risk. Notably, more than 42% of patients aged 60 to 69, and those with a SCORE2-OP risk score of 20% or higher, were found to have cardiac pathology. This goes to support the idea that focused screening in these groups is worthwhile. Literature data also supports this idea. For instance, prior AI-FOCUS cohorts studied by Motazedian et al. [30] have shown that even novice operators achieved a 93% sensitivity and 92% specificity in detecting LVEF < 50%, with excellent inter-operator consistency. This proves as an advantage especially for frontline clinicians, who can perform triage and lower the burden of over-specialists.

Even though men had a slightly higher rate of findings, the AI tool performed consistently well for both men and women. Likewise, the ESC’s validation [31] of SCORE2-OP across a way larger cohort of patients demonstrated robust predictive value (C-index 0.63–0.67) and helped identify individuals aged 60–69 or with SCORE2-OP ≥ 20% as having over 42% likelihood of cardiac findings. Therefore, AI-assisted FOCUS maintained strong accuracy across genders.

In our subgroup analyses, AI-assisted FOCUS demonstrated consistently high diagnostic accuracy across age, sex, and SCORE2-OP risk strata. The diagnostic yield was particularly strong in patients aged 60–69 years (40.6% prevalence of cardiac pathology) and in those with SCORE2-OP risk ≥ 20% (≥42% prevalence). Although men had a slightly higher prevalence of cardiac abnormalities than women, the performance of AI-FOCUS remained robust across sexes. These findings suggest that while AI-FOCUS is broadly applicable in primary care, its greatest clinical value may be realized in older, higher-risk patient groups where the prevalence of undiagnosed pathology is highest.

Table 7 shows the comparison between out trial and the latest and most relevant trials on the same topic.

Altogether, these results suggest that AI-supported FOCUS is a practical and reliable addition to primary care. It can help catch cardiac issues earlier and guide timely referrals to specialists.

### 4.2. Resource Optimization

Our results suggest that AI-FOCUS [33] can significantly optimize healthcare resource utilization. Early detection of previously unrecognized cardiac pathology (32.9% of patients) allowed for timely and appropriate cardiology referrals. Conversely, 40% of FOCUS-negative patients avoided unnecessary specialist evaluation, reducing referral burden and healthcare costs These findings that align with prior WHO reports on cost-saving potential of mobile cardiac diagnostics.

Using Romanian health system data [1], the average cost of echocardiography is €110 per exam. By avoiding unnecessary referrals in 40% of patients (712/1780), AI-FOCUS could save approximately €78,000 per 1000 patients screened (~€430 per 100 patients). This exploratory estimate highlights the potential for significant cost savings in resource-limited systems.

Consistency, as in reproducibility, is also an important factor in optimizing resources. Fortunately, AI has been shown to help in that matter. In a large multicenter study by Kagiyama et al. [32], AI-assisted LVEF measurements using handheld devices showed excellent reproducibility: intraclass correlation coefficient = 0.90 overall, with 0.92 for novices and 0.85 for experienced users. Agreement with formal echocardiography remained high (weighted κ = 0.83), sensitivity was 92.8%, and specificity 92.3% for detecting LVEF < 50%. This means that, in short, no matter who was using the device, the LVEF readings were reliable and consistent.

### 4.3. Limitations

There are several important limitations to consider when interpreting our findings. Although the general practitioners (GPs) in our study underwent structured training in focused cardiac ultrasound (FOCUS), the variability in training quality and experience across different clinical settings may limit the generalizability of our results. Operator skill remains a critical factor, particularly when performing scans in patients with obesity or chronic obstructive pulmonary disease (COPD), where image quality is often compromised. As highlighted by Moore et al. [34], the effectiveness of point-of-care ultrasound (POCUS) is strongly influenced by the training and experience of the user, and this variability can significantly affect diagnostic outcomes in real-world practice.

Another key limitation relates to the demographic characteristics of our study population. Our research was conducted in a single geographic region and involved a predominantly Caucasian cohort. While this provides internal consistency, it restricts the broader applicability of our findings. Results are uncertain if we were to generalize among populations and medical systems. Algorithm bias cannot be excluded, and external validation is needed across diverse populations. As Bullock-Palmer et al. have emphasized in their multicenter cardiac imaging research [35], diverse population sampling is essential to validate diagnostic tools like FOCUS across varied demographic and clinical environments.

The training pathway was designed for screening-level competence; our study does not claim parity with comprehensive specialist echocardiography. Errors occurred primarily in borderline or subtle findings, such as mild valvular insufficiency, borderline LVEF reductions, or minimal pericardial effusions. These limitations highlight the need for cardiologist confirmation in cases where AI-FOCUS suggests borderline or equivocal findings.

Lastly, while our AI-assisted system performed well in estimating left ventricular ejection fraction (LVEF) and identifying basic valvular abnormalities, its performance remains limited in more advanced echocardiographic evaluations. Specifically, automated assessments of diastolic dysfunction and the quantification of valvular regurgitation are areas where current AI models fall short. These tasks still require expert input from trained cardiologists. This limitation is consistent with findings from Sengupta et al. [36], who note that while AI is increasingly reliable in core cardiac metrics, more complex interpretive tasks continue to benefit from human oversight and clinical judgment.

### 4.4. Future Perspectives

In order to ensure the reliable, cost-efficient and reproducible long-term use of AI in POCUS, there are some fields which either show a promising future or require further investment and research. Firstly, standardizing training and certification processes is essential to minimize operator variability. A recent pilot program in primary care [37] introduced a structured AI-focused cardiac ultrasound curriculum that substantially improved novice users’ diagnostic skills, with diagnostic accuracy rising from 70% to over 90% after training. Moreover, a UK-based community screening program using handheld cardiac ultrasound (HHCU) by Mitchell et al. [38] reported its cost-effectiveness, with screening costing only approximately £43 to perform.

A rigorous validation across diverse populations and healthcare systems is vital to confirm worldwide standardization. Fortunately, recent studies show very good prospects. A recent multicenter validation study in Japan [32] involving two hospitals demonstrated that AI-POCUS successfully estimated LVEF in 182 patients, showing excellent agreement with standard echocardiography (intraclass correlation coefficient 0.81, sensitivity 85%, specificity 81%). It is therefore important to acknowledge AI-based POCUS’ broad clinical applicability beyond specialized centers.

Moreover, integrating AI-FOCUS into telemedicine platforms can dramatically increase access to quality cardiac care in underserved and rural areas. In a multicenter validation of AI-guided lung ultrasound [39], non-experts using AI achieved diagnostic-quality images in over 98% of cases, matching expert performance. Therefore, AI-powered remote guidance could similarly support cardiac imaging via telemedicine and potentially reduce geographic differences in access to specialized doctors.

Concerning areas in which AI still has a lot to learn, future studies should invest in expanding AI capabilities to encompass diastolic function, valvular quantification, and ultrasound in order to create a more complete cardiopulmonary evaluation. In this respect, advances in deep learning, as described by Tsampras et al. [40] in their recent review, have already enabled multi-agent AI educational tools, pointing the way toward more elaborate AI-assisted platforms that optimize cardiac assessment without requiring expert oversight.

## 5. Conclusions

This study demonstrates that artificial intelligence–assisted Focused Cardiac Ultrasound (AI-FOCUS), when performed by trained family physicians, can achieve high diagnostic accuracy, excellent inter-rater agreement, and meaningful clinical impact in a primary care setting. With an overall diagnostic accuracy of 94.33% and a Cohen’s kappa of 0.88 compared to cardiologist-performed echocardiography, AI-FOCUS significantly improves the early detection of cardiac abnormalities, particularly left ventricular systolic dysfunction and valvular disease. Importantly, AI-FOCUS enabled the timely referral of 32.9% of patients with previously unrecognized pathology while safely reducing unnecessary referrals in 40% of those with negative scans—an outcome with clear implications for cost savings and healthcare system efficiency.

Despite its promise, the successful implementation of AI-FOCUS requires standardized training pathways, validation across diverse populations, and continuous AI refinement to handle more complex assessments like diastolic dysfunction and advanced valvular pathology. Future integration into telemedicine platforms could further expand access, particularly in rural or underserved areas where cardiology services are limited.

To summarize, AI-assisted FOCUS represents a scalable and reliable tool for improving cardiovascular risk stratification and diagnostic triage in primary care. It has the potential to fill a critical gap between the limitations of traditional physical examination and the resource demands of comprehensive echocardiography. As cardiovascular disease remains a leading cause of morbidity and mortality worldwide, incorporating AI-FOCUS into routine practice may contribute meaningfully to early diagnosis, better care coordination, and a more efficient use of healthcare resources.

## Figures and Tables

**Table 1:** Diagnostic performance of AI-FOCUS. Counts: TP = 526, FP = 42, TN = 1153, FN = 59 Abbreviations: PPV, positive predictive value; NPV, negative predictive value; CI, confidence interval; κ, Cohen’s kappa.

Metric	Value (%)	95% CI Lower	95% CI Upper
Accuracy	94.33	93.15	95.35
Sensitivity	89.91	87.18	92.23
Specificity	96.49	95.28	97.46
PPV	92.61	90.29	94.41
NPV	95.13	93.88	96.14

**Table 2:** Cardiac pathology distribution. LVEF, left ventricular ejection fraction.

Pathology	Count	Prevalence (%)
Valvular abnormalities (mitral regurgitation and others)	246	42.0
LVEF < 50%	164	28.0
Pericardial effusion	70	12.0

**Table 3:** Logistic regression predictors of cardiac abnormality. OR: odds ratio; CI: confidence interval.

Predictor	Odds Ratio (OR)	95% CI	*p*-Value
AI-assisted LVEF < 50%	6.05	4.92–7.45	<0.0001
Valvular abnormalities	4.05	3.11–5.28	<0.0001
Hypertension	3.67	2.81–4.79	<0.0001
Pulmonary B-lines	3.02	2.15–4.23	<0.05

**Table 4:** Cardiac pathology prevalence by age group. Prevalence, proportion with confirmed cardiac pathology by subgroup.

Age Group	Total Patients	Patients with Pathology	Prevalence (%)
40–49	320	72	22.5
50–59	480	144	30.0
60–69	620	252	40.6
70–75	360	117	32.5

**Table 5:** Cardiac pathology prevalence by gender. Prevalence, proportion with confirmed cardiac pathology by subgroup.

Gender	Total Patients	Patients with Pathology	Prevalence (%)
Male	961	340	35.4
Female	819	245	29.9

**Table 6:** Cardiac pathology prevalence by SCORE2-OP risk strata. Prevalence, proportion with confirmed cardiac pathology by subgroup.

SCORE2-OP Risk Strata	Total Patients	Patients with Pathology	Prevalence (%)
10–14%	500	110	22.0
15–19%	600	180	30.0
20–24%	400	170	42.5
≥25%	280	125	44.6

**Table 7:** Comparison between our trial and the latest others in the same domain.

Study	Setting	Sample Size	Main Outcome	Accuracy/Sensitivity/Specificity (or Relevant Metric)	Key Limitation	Novelty of Current Study
Motazedian et al., 2023 [30]	Cardiology ward	300	AI-FOCUS LVEF	93%/93%/92%	Small sample, limited pathologies	—
Papadopoulou et al., 2024 [17]	Oncology staff (chemotherapy)	120	AI-FOCUS LVEF	sensitivity of 95% and specificity of 94% for the cardiologist	Specialist oncology cohort, not primary care	—
Kagiyama et al., 2024 [32]	Multicenter (Japan)	182	AI-handheld LVEF	AUC 0.92, Sens 85%, Spec 81%	Small multicenter sample, limited scope	—
Current Study	Primary Care (Romania)	1780	AI-FOCUS in multiple pathologies	94.3%/89.9%/96.5%	Single-center, Caucasian population	Largest prospective study in primary care; multiple pathologies evaluated

## Data Availability

Data will be made available for valid written requests addressed to the corresponding authors.

## Supplementary Materials

The following supporting information can be downloaded at https://www.mdpi.com/article/10.3390/healthcare13212726/s1; Table S1. Checklists of model features, results and limitations. Table S2. Hyperparameters and overfitting handling.
