# Supplementary material for: Diagnostic Accuracy of AI-Assisted Focused Cardiac Ultrasound (FOCUS) in Primary Care
Source: Healthcare (Basel). 2025 Oct 29;13(21):2726. doi: 10.3390/healthcare13212726 (PMC12610991; doi:10.3390/healthcare13212726)
Supplement: Supplementary file 1 [file healthcare-13-02726-s001.zip › healthcare-3880068-supplementary.pdf]

Supplemental Tables:

Table S1. Checklists of model features, results and limitations.

Category	Details
Population	1780 patients, age 40–75, SCORE2-OP ≥10% or symptomatic
Training of physicians	6 months training, >50 supervised scans, competency exam
AI platform	Wis+ AI (Sonoscape-P60) – automated LVEF + speckle-tracking
Primary outcomes	Detection of LVEF <50%, valvular disease, pericardial effusion
Diagnostic performance	Accuracy 94.3%, Sensitivity 89.9%, Specificity 96.5%, κ=0.88
Failure conditions	Mild regurgitation, borderline LVEF 45–50%, small pericardial effusions
Comparisons to prior work	Improves on smaller studies (Motazedian 2023, Papadopoulou 2022, Kagiyama 2024)
Limitations	Single-center, Caucasian cohort, limited advanced echo (diastolic dysfunction, quantitative regurgitation assessment not supported by AI)

Table S2. Hyperparameters and overfitting handling.

Component	Details
Model type	Convolutional Neural Network (CNN) – proprietary Wis+
Input data	Standard echocardiographic cine loops (apical 4-chamber, parasternal views)
Segmentation method	Automated endocardial border detection + Simpson's biplane method
Frame rate	30 frames per second
Output metric	Left ventricular ejection fraction (%)
Threshold for abnormality	<50% classified as abnormal
Overfitting mitigation	Pre-trained and validated by manufacturer; in this study, 5-fold cross-validation confirmed robustness across patient subsets
